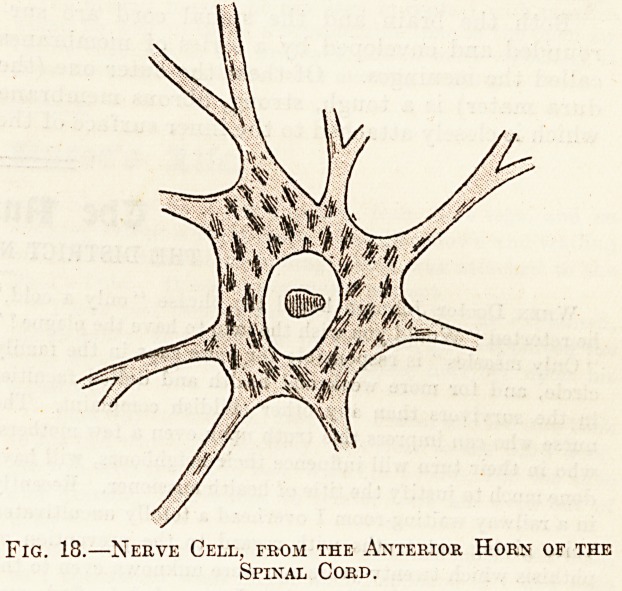# "The Hospital" Nursing Section

**Published:** 1906-08-18

**Authors:** 


### The Hospital.

### Nursing Section.

Contributions for "THE HOSPITAL," should be addressed to the EDITOR, "THE HOSPITAL" NURSING SECTION, 28 & 29 Southampton Street, Strand, London, W.C.

No. 1,039.—VOL. XL. SATURDAY, AUGUST 18, 1906.

### Notes on News from the Nursing World.

### THE QUESTION OF PREVIOUS TRAINING.

IN the course of the interview between the matron of Birmingham General Hospital and our Commissioner, which is reported in our columns to-day, Miss Jones referred to many topics of interest, including the importance of nurses, desiring to become matrons, understanding the details of housekeeping, the usefulness of special experience, and the question of previous training. Some of our readers will, doubtless, be glad to know that Miss Jones rather likes to have probationers who have had previous experience, providing always that they enjoy a good record. She also alludes to the fact that candidates of twenty-one are now admitted if they are pronounced on examination to be physically fit. The rules were altered last year to the latter effect, and the matron of the Birmingham General Hospital does not see why women who wish to occupy their time before their final training, should not go in for children's or fever training. She admits that she herself has found her own fever training extremely valuable throughout her career.

### MIDWIFERY IN POOR-LAW INFIRMARIES.

WE notice that a contemporary, referring to our comments on the decision of the Privy Council to remove the training of midwives in poor-law institutions from the jurisdiction of the Central Midwives Board to the Local Government Board, describes THE HOSPITAL, as "one of the strongest defenders of the Midwives Board." On this ground, it is evidently surprised that we did not condemn the action of the Privy Council. But we have simply defended the Midwives Board when we have thought that it was unfairly attacked, as we have not hesitated to differ from it when we have believed it to be in the wrong. There are obviously objections to the establishment of a second authority for the licensing of midwives, but we presume that the Privy Council are satisfied that they are less serious than the perpetuation of a condition of affairs which has acted to the detriment of some of the most important poor-law infirmaries in the country.

### AN INCIDENT WHICH OUGHT TO BE IMPOSSIBLE.

THE incident headed, "A Fright in the Night," which is related on another page, supplies a lesson to hospital authorities which should not be lost sight of. No night nurse can possibly get through her work satisfactorily, doing her duty to her patients and herself, without getting a meal which necessitates her withdrawal from the wards for a short time. In this particular instance, our contributor, to obtain the requisite meal, had to leave unattended thirty-five surgical and accident cases occupying two different wards, including three patients who had recently been operated on. Fortunately the adventure of the male patient who was the cause of the nurse's fright had no disastrous result. If it had been otherwise, the blame would not have attached to the nurse but to the hospital authorities. It is bad enough that one ward should have to be left unattended while the nurse in charge is in the other; but under any circumstances a nurse ought not to be put in the position of having to choose between leaving a landing of two wards without attendance or going for an undue length of time without food.

### NURSES AND TRAM FARES.

IT is proposed by a Sunderland ratepayer who describes himself as a working man, that the nurses in the town should be allowed to travel by the trams free of cost. He has noticed that policemen jump into the cars and ride without payment, while he has seen nurses trudging along the road on their way to the relief of the sick poor, and he does not like the contrast. We appreciate his chivalry, but we do not think that, generally speaking, nurses claim to be treated in the same manner as policemen. The latter are the officials in the employ of the representatives of the ratepayers; but nurses, even if they be district nurses helping individually to keep down the rates, are not. Moreover, it is not desirable that any person, merely because she happens to be wearing the uniform of a nurse, should be permitted to use the trams free of expense. In the case of a district nurse, however, her committee should take care that she is not under the necessity of walking long distances to see her patients or paying the fare out of her scanty earnings.

### INSUFFICIENT STAFF AT EXETER INFIRMARY.

AT the last meeting of the St. Thomas's Guardians Mr. Preston Thomas, Local Government Board Inspector, stated that he was strongly of opinion that the nursing staff at the Infirmary was insufficient. He pointed out that a good many of the 70 cases on the books required constant attention by night and day. Subsequently the Chairman admitted that, while there was only one nurse to every 32 patients in the Infirmary under the charge of the Guardians, there was usually one to every 15, and he expressed a desire to engage two additional nurses. Mrs. Edwards suggested that a man should be engaged as one, but Mr. Preston Thomas said that male nurses had not always proved satisfactory, and the suggestion was set aside.

There may, of course, be circumstances in which the substitution of male for female nurses is desirable; but the exception only proves the rule.

### THE INJURED AT THE HANDCROSS ACCIDENT.

The valuable services and kindness shown by the medical staff and nurses to the sufferers from the disaster at Handcross Hill have been recognised by the owners of the Vanguard motor-bus which met with so deplorable an accident. They have sent a cheque for £50 to the Crawley Cottage Hospital, to which, as our readers know, so many of the injured were taken. There are now only two patients in the hospital, and as these are making excellent progress towards recovery, we may congratulate the matron upon the termination of an exceptionally anxious time.

### A PIN AS A CREDENTIAL.

There is the same difficulty in the United States about uniforms as there is in England, notwithstanding the partial adoption of registration. An American nurse, in her despair at the hopelessness of affording protection to the fully-trained nurse, suggests the adoption of "a State or national pin, to be worn always when on duty." She says that she should not feel labelled or hampered with it, but rather proud that she should be able to earn it. But we are afraid that the introduction of a pin as a badge would hardly suffice to enable the American public to distinguish between the trained and the inexperienced nurse. It seems a poor sort of credential of capacity.

### CENTRAL MIDWIVES BOARD EXAMINATION.

The list of successful candidates in the examination of the Central Midwives Board held on August 1 shows that 245 candidates were examined. Of these 192 passed, and the percentage of failures was 21.6. The successful candidates included 30 trained at the Maternity Charity, Plaistow; 23 at the General Lying-in Hospital; 12 at the Salvation Army Maternity Hospital; 7 at Guy's Institution for Trained Nurses; 7 at Queen Charlotte's Hospital; 7 at the London Hospital; 6 at the British Lying-in Hospital; and 6 at the City of London Lying-in Hospital. There were several successful candidates trained in obscure institutions, but only two in Poor-law institutions, and these were contributed by Greenwich Union Infirmary.

### OPENING OF A NEW TENNIS-LAWN AT THE BIRMINGHAM CITY HOSPITAL.

The authorities of the Birmingham City Hospital have provided the sisters and nurses in their service with an excellent tennis-lawn. The lawn was formally opened last Wednesday week, the event being celebrated by a garden-party. The tennis-set was kindly presented by the medical superintendent.

### DISTRICT NURSING IN IRELAND.

The third annual report of Lady Dudley's scheme for the establishment of district nurses in the poorest parts of Ireland is highly satisfactory as to the work done, but it also indicates the pressing need of further appointments. At present there are 14 fully trained nurses employed in the various districts, and their courage and devotion, their patience and kindness in ministering to the sick poor seems to have excited enthusiasm everywhere. Lady Aberdeen, who has entered with spirit into the movement originated by her predecessor, will not, we think, appeal in vain for financial assistance to enable her to extend the scope of the enterprise.

### A NOTABLE INSTITUTION AT SYDNEY.

The opening, a few weeks ago, of a district nursing branch in association with the St. Margaret's Hospital for Women at Sydney, New South Wales, is the development of a notable institution. The hospital, then called St. Margaret's Maternity Home, was founded by Mrs. Abbott in March 1894 with two beds, and without capital, for love of charity. The original idea was to furnish a home for unmarried girls who had fallen for the first time, but after a little while the scope was enlarged, and poor married women were taken in too. In 1894 thirty-two maternity cases were attended and three nurses trained. Last year the number of cases attended was nearly 400, and the number of nurses trained was twenty-six. A large number of surgical operations were performed at the hospitals and in homes of the patients, while 1,200 women consulted the honorary medical staff. There has always been a difficulty in obtaining funds, but the unique feature is that there have never been any salaries to pay. Mrs. Abbott, who still acts as matron, has given her services throughout, and so have the sisters and nurses. The principal source of income has not been subscriptions, but the fees received for instruction in midwifery. The course is one of six months, and each probationer pays twenty-five guineas for board, lodging, and training. As the teachers make no charge, the balance of these fees, after deducting the expense of housing and feeding, goes towards the maintenance of this excellent charity.

### PROGRESS AT GRANTHAM.

At the annual meeting of the Grantham Victoria Nursing Association a very satisfactory report was read and adopted. Mention was made of the fact that during the past four years there had been no deficit, and that a reserve sum had been formed. During last year legacies to the extent of £1,000 were left by former generous supporters, and the Mayor, in congratulating the members of the Association upon its flourishing condition, said that there was no doubt that the efforts of the committee to carry it on successfully were appreciated. An address was subsequently delivered by Miss Amy Hughes, the general superintendent of Queen Victoria's Jubilee Institute, and it was decided to ask for an additional Queen's nurse to be sent from London, on account of the increased work in Grantham.

### SHORT ITEMS.

Miss A. A. Mackenzie and Miss F. Needham have been appointed nursing sisters in Queen Alexandra's Military Nursing Service for India.— Among the numerous bequests of the late Mr. Edward Rawlings is £500 to the Royal National Pension Fund for Nurses.

### The Nursing Outlook.

"From magnanimity, all fears above;
From nobler recompense, above applause,
Which owes to man's short outlook all its charm."

### PRACTITIONERS AND NURSES.

If we look back to the sixties and seventies we find that the relations between the practitioner and the nurse approximated to that of master to servant. These relations were no doubt governed to a great extent by the fact that the nurse of those days was mainly, if not entirely, drawn from the servant class. The surgeon of those days selected women in whom he had confidence and taught them to nurse his cases in accordance with rules which he laid down, so that the practitioner was able to rely upon his methods being strictly pursued in his absence. In this way the nurse was largely, and indeed mainly, dependent upon the goodwill of the practitioner for the maintenance of her position and the making of a livelihood by nursing. At the end of the sixties and during the subsequent eight or ten years strenuous efforts were made by the authorities of St. Thomas's Hospital, London, and the managers of the Queen's Hospital, Birmingham, to induce a more educated and better class of women to take up hospital nursing. The difficulties, largely due to prejudice against the hospitals from the high mortality and relative inefficiency of such establishments in those days, which had to be overcome, were immense. Mrs. Wardroper and Mr. Burdett, however, acting in co-operation, by mutual encouragement and persistence, succeeded in securing a number of younger women to be trained on a definite system in the art of nursing, and gradually a change for the better was brought about.

Current hospital opinion supported the view that widows and women who had attained middle age were the most suitable for work in a hospital. It took some years to modify this opinion, and to prove that a higher moral standard and a much greater efficiency could be attained in the nursing department by employing much younger women of education and high character. It is not easy for the matron of to-day, who is overwhelmed with applications for the post of probationer, to realise that less than forty years ago, out of a hundred applicants obtained with great difficulty, less than ten per cent. were found to be capable of completing a year's training in the hospital and so qualifying for permanent employment as a nurse. To-day everything is changed. The number of applications received by a matron of a big hospital is practically unlimited, and, although the wastage is still considerable, there is no difficulty in procuring any number of suitable women for training in a hospital. The tendency of the day is to exclude all applicants except those who have had a good general education, and for the most part the superior servant class of probationer is not favoured, though we think that those matrons who assume this attitude may thus exclude an army of the best type of nurses from the profession.

It is only natural and proper that as the standard of education for nurses has been raised, that the claims of the nurses themselves to higher emoluments and a recognised position have increased too. We have always held, however, that character in a nurse is the most material of all considerations from the patient's and practitioner's point of view, and that, given the requisite technical knowledge, womanly qualities are the chief essentials to success. It is greatly to the credit of the whole nursing body that what we may describe as the radical section of nursing opinion has shown a tendency to diminish rather than increase during the last few years. Certain active spirits, known as the stage army, have made enough noise to justify the belief that they have infinitely more weight and importance than they really possess. The claim they have put forward on behalf of the nurses to paramount control on any General Nursing Council which may be set up by Parliament has not won the assent of the great body of British nurses. Advocates of this principle have denounced the British Nurses' Association and done their utmost to destroy that body, on the ground that the Registration Bill introduced into Parliament under its auspices does not give paramount representation to nurses. The hollowness of this attack is exposed by the action which those same advocates have taken in regard to the decision concerning State registration arrived at by the British Medical Association.

The British Medical Association has decided to approve the recommendation of the Parliamentary Select Committee that there should be State registration of nurses, and has expressed the opinion that on any Central Council or board appointed the medical profession and the nursing profession should be adequately and directly represented. Had the resolution been carried in these terms there might have been some ground for claiming that it supported the stage army claim that the nurses should elect a majority of the General Nursing Council. So far from this being the fact the practitioners present at the annual meeting of the British Medical Association carried an amendment to the effect that the representation of the medical profession on the Central nursing body should be at least one-half of the number of the members of that body. What then becomes of the claim of the advanced party that the action of the British Medical Association supports their view, that the paramount control of the registration council should be in the hands of nurses directly elected by themselves to represent their interests?

### The Care and Nursing of the Insane.

By Percy J. Baily, M.B., C.M.Edin., Medical Superintendent of Hanwell Asylum.

### I.—ANATOMY AND PHYSIOLOGY.

(*Continued from page* 259.)

### 8. The Nervous System.

We have seen that the various organs or systems of organs in the body all depend very much one upon the other—each system has to do its own particular work for the common welfare of the whole organism. The blood is the agent through which the various organs of the body are brought into relation with one another, and which is affected directly by all the organs which we have up to the present been discussing—they all either give to it or take from it, and hence we cannot say that one system of organs is more important in the whole economy than another. The remaining system which we have to deal with is, however, in the nature of its functions, so high above all the rest, and occupies such a commanding position over them all, that we may certainly regard it as being the all-important one in man. This is the nervous system. It controls and regulates the functions of all the other organs. It originates all muscular action. It governs and controls the movements of the heart and blood-vessels. It is through the influence of the nervous system that the various digestive juices are secreted, and that the movements of the alimentary canal are regulated. The act of respiration, the secretion of the urine and sweat, the growth of the various tissues of the body, and the assimilation of the food from the blood and the proper repair of damaged and worn-out tissues, the maintenance of the proper temperature of the body, etc., etc., all depend upon the healthy working of the nervous system. It also receives and interprets impressions or sensations from without the body, whereby we are brought into relation with the outer world and our fellow-creatures, and from within the body, whereby we are made cognisant of the condition of our own organs and tissues, and it is the seat of the mind.

The nervous system is built up of two kinds of tissue, which are called respectively the grey matter and the white matter. These two different kinds of tissue can easily be distinguished from one another by the appearance they present to the naked eye, and when examined with a microscope their differences are still more marked. As we shall see presently, the grey matter and the white matter occupy certain definite parts of the nervous system, and are not indiscriminately intermixed. Both forms of nervous tissue consist of certain nerve elements, which are bound together and supported by a delicate connective tissue called neuroglia. Those parts which consist of grey matter are spoken of as nerve centres, and their distinguishing peculiarity is that they are built up very largely of nerve cells (fig. 18). These nerve centres, however, contain many nerve fibres as well as nerve cells, the fibres passing from one cell to another, and thus connecting them with each other. The cells exist in different parts of the grey matter of the nervous system, in all varieties of shapes and sizes, the most typical being somewhat large (larger than a red blood corpuscle), irregular, more or less angular masses of protoplasm. From

Fig. 18.—Nerve Cell, from the Anterior Horn of the Spinal Cord.

their corners or angles there pass delicate branching nerve fibres to or towards other similar cells; while from one side of the cell there arises a larger unbranched process or fibre which, soon after leaving the cell, becomes covered with a white sheath. This fibre, with many other similar ones, goes to build up one of the cranial or spinal nerves, as we shall presently see. The white matter of the nervous system is composed entirely of these white unbranched processes from the nerve cells (held together and supported by neuroglia). Both the grey and the white matter are, of course, abundantly supplied with blood-vessels, for the nervous system cannot carry on its functions without an abundant supply of pure arterial blood.

Briefly, then, the nervous elements of the grey matter are nerve cells as well as nerve fibres, while those of the white matter are nerve fibres only. We shall presently see how these two varieties of nerve tissue are arranged in our bodies.

For the purposes of description the nervous system is said to comprise two sets of nerve centres and nerve fibres.

1. The cerebro-spinal or central nervous system.
2. The sympathetic system.

It must not be imagined that these are distinct from one another, for they are intimately connected together, and the dominating member of the partnership is the cerebro-spinal system, the sympathetic system receiving guiding influences, or, as we may say, instructions from the brain and spinal cord.

I. *The Cerebro-Spinal Nervous System.*—This comprises the brain and spinal cord, together with the nerves which arise from them. Those nerves which arise directly from any part of the brain are called the cranial nerves, while those which come from the spinal cord are called the spinal nerves.

The brain occupies the cavity of the skull, while the spinal cord is contained within the spinal canal in the backbone. The cranial nerves leave the skull by passing through holes in the bones which form its walls, while the spinal nerves pass out from the spinal canal between the different vertebræ.

Both the brain and the spinal cord are surrounded and enveloped by a series of membranes called the meninges. Of these the outer one (the dura mater) is a tough, strong, fibrous membrane which is closely attached to the inner surface of the bones of the skull, but lies loosely within the spinal canal. The inner membrane, called the pia mater, is closely attached to the surface of the brain and spinal cord, which it completely envelopes. It is a very thin and delicate membrane, but contains a large number of blood-vessels. Between the dura and the pia there is a certain amount of fluid which serves to support the brain and in which it, as it were, floats.* When any of these membranes or meninges become inflamed the disease which results is called meningitis.

* There is a third membrane called the arachnoid lying between the dura and pia, but for our present purpose, and for the sake of simplicity, it is unnecessary to include this in the general description.

### The Nurses' Clinic.

### THE DISTRICT NURSE AND MEASLES.

When Doctor Johnson heard the phrase "only a cold," he retorted "Would you wish the man to have the plague?" "Only measles" is responsible for more gaps in the family circle, and for more weakened health and dulled faculties in the survivors than any other childish complaint. The nurse who can impress this truth upon even a few mothers, who in their turn will influence their neighbours, will have done much to justify the title of health missioner. Recently in a railway waiting-room I overhead a totally uncultivated voice giving out truths with regard to the prevention of phthisis which twenty years ago were unknown even to the liberally educated. The voice, I was glad to find, was specially strong as to the danger of allowing children "who may be quite well, but a bit weakly" to visit any house where there was a consumptive patient.

With the exception of influenza, measles is probably the most infectious of all fevers, and there is some excuse for tired mothers who say impatiently, "They've got to have it." It must be acknowledged that in crowded towns there is not much hope of entire immunity, and the district nurse should devote most of her energy to trying to persuade parents that the longer the attack is postponed the less injurious it will be, and the older the child the easier it will be to take due care of it during the illness. It is in carrying a child too young to be left alone from room to room that most of the complications originate. An unfortunate and entirely mistaken prejudice exists that the younger the child is the more lightly it escapes. This disastrous belief is not only found among the poor; I have known an unhappy middle-class baby, aged six months, living in a large and rambling country house, where it could easily have been kept apart, deliberately shut up with its three elders of five, seven, and eight, who were suffering from measles. Their illness was of the mildest description, but the baby nearly died, and remained sickly and unhealthy throughout its entire childhood.

The majority of fatal cases occur before the end of the second year, and more than four-fifths of the children who die from measles and its complications are under four years of age. It is slightly more dangerous for boys than girls, and especially dangerous for those of a tuberculous tendency. The chief points to be dwelt on by the district nurse are: The child must be kept in bed in one room at a temperature of not less than 60° F., and should remain in bed until his temperature has been normal for a week. Flannel nightshirts should be worn, and if the patient is vigorous and restless and given to hopping in and out of bed, these should be replaced by pyjamas and thick bed socks. As the eyes are usually weak, the light must be subdued. Even in mild attacks and favourable circumstances the child should be kept indoors for four weeks from the beginning of the illness, and in the case of damp, raw weather, or a naturally delicate subject, a considerably longer time must be allowed before the child goes out, and specially warm clothing and nourishing food must be provided.

When one considers that among the possible complications and sequelæ of measles must be counted diphtheria, bronchitis, pneumonia, phthisis, inflammation of the ear, abscess of the brain, ophthalmia, and dysentery, it cannot well be denied that it is a disease that requires far more careful nursing than it usually receives. The most deadly and frequent complication is broncho-pneumonia. In children under three it is almost invariably fatal.

Purgatives may be needed, but owing to the tendency to diarrhœa, and even dysentery, only mild ones can be used, such as castor oil, or a warm water enema with two teaspoonfuls of castor oil may be given. Bleeding from the nose often occurs, and (unless severe) is a natural relief that should not be interfered with. It may be necessary to give a warm bath to bring out the rash, but as a rule it is safer to keep the child quiet and leave the matter to nature.

The period of incubation varies, but is usually about fourteen days; throughout this time the child is in a vaguely ailing condition, fretful and inclined to tears. The rash generally appears about the fourth day of the disease; on the fifth or sixth day it is at its height; on the sixth or seventh day it disappears from the face, and a day or two later from the body; in severe cases it lasts longer. The rash shows first on the face, and is always more raised and decided there than on the trunk, to which it spreads in the course of a few hours. It consists at first of round, red spots, which on the head and face soon join into a flush which varies from a dusky, unwashed looking tinge to a deep red. There is a characteristic effluvium by which experienced persons, whether nurses or not, can recognise the disease. It must always be impressed upon mothers that one attack does not secure immunity. I have known a child of eleven seriously ill with what was undoubtedly her third attack, but probably this was a rare instance.

In addition to the constitution of the child and the apparent severity of the fever, the nature of the local epidemic as a whole must be considered. In early days measles were regarded as closely resembling small-pox and more than rivalling it in deadliness, and although in Europe at the present day the outbreaks are never of this virulent and destructive nature, the character of the disease as an epidemic varies greatly, and even within the last forty years

**THE NURSES' CLINIC**—*Continued.*

it has several times been known to occasion a higher rate of mortality than scarlatina. The most horrible outbreak on record was at Fiji in 1875, when 26 per cent. of a population of 150,000 died within four months. The longer any population has been entirely free from measles, the more severe the outbreak will probably be when it takes place; but with regard to the high death-rate it must be remembered that when four or five persons are ill in one house they are almost inevitably nursed with less care than if there were only one patient.

The early symptoms of measles (often overlooked on account of their unobtrusive nature, and especially in children of twelve or more) are a slight dry cough, eruption, sneezing, irritation of the eyes, and a rise of temperature. Infection can be conveyed by blood, tears, or other secretions, but it is not definitely known whether the bacteria and micro-cocci found in the blood are the cause or the consequence of the disease. The vitality of the infectious principle, whatever it may be, is probably weak; most doctors are of opinion that a day spent out of doors on a windy moor, or by the seaside, is enough to purify infected clothing.

The district nurse who has been in contact with measles would be wise to spend a considerable time in the open air before engaging in her ordinary duties, more especially if these have any connection with monthly nursing.

### Incidents in a Nurse's Life.

### A FRIGHT IN THE NIGHT.

I was night-nurse of a male surgical and accident landing, consisting of two wards—in all thirty-five beds—and was, therefore, always busy when on duty.

There had been three operations during the day, and a fractured femur had been admitted. On the day before a colotomy had been done, a Paul's Tube being left in, and I had also under my charge a strangulated hernia, who had only that evening left off vomiting.

It was, therefore, not to be wondered at that it was after one before I could get what was called my midnight meal. I was hurrying over it, unable to feel easy for a moment out of the ward, with so many of the patients in such a critical condition, when I heard a noise as of bare feet just outside the kitchen-door. For a second I held my breath in an agony of apprehension, and watched, the feeling that something was about to appear becoming intensified every second.

A final shuffle of feet and I saw—a tall, gaunt figure, white-faced, white-haired, with trembling, fumbling hands stretching out as if for support; lean, bare legs, and no clothing except a night-shirt. Hanging down and trailing behind was the rubber tubing which was attached to the Paul's Tube. It was the colotomy patient

"I was just looking about for a drink," he explained, incoherently, as I took him by the arm, and supported the tube which must have been dragging horribly upon his intestines.

"This way for a drink, Daddy," I said, and led him back to his bed which, fortunately, was close to the ward door. He was soon settled in bed, with hot bottles, and the tube and dressing, marvellous to relate, were not even out of place!

For a few moments I trembled as much as the old man, as I speculated what might have happened if I had been busy in the other ward, or if the rubber tubing had caught in anything.

As far as we could tell, the old man took no harm from his adventure. He died some weeks later of the malignant disease from which he was suffering.

### The Nurses of the General Hospital, Birmingham.

### INTERVIEW WITH THE MATRON. BY OUR COMMISSIONER.

Among leading nurse-training schools Birmingham General Hospital occupies a foremost place, worthy of the splendid building itself and of the metropolis of the Midlands. When I saw Miss M. E. Jones, the Matron, she suggested that before we discussed the nursing, we should look round some of the wards, the Nurses' Home, and other features of interest in the institution. I was particularly impressed by the completeness and the excellence of the arrangements made for the commissariat. The kitchens, which are extremely commodious and lofty, are at the top of the building.

### The Housekeeping.

"We pay great attention to this department," said the Matron. "Formerly, the housekeeper was not a trained nurse, but soon after I came here the Committee asked me to be responsible for it, and since then there have been very few complaints from either the patients or the nurses respecting the food. The department is in charge of the assistant matron, under my supervision, and the experience I think is not only good for her, but also for the sisters. As you are doubtless aware, our sisters often become matrons of other institutions, and I do not see how they could undertake the obligation of feeding a number of people unless they knew all about food. The possession, or otherwise, of such knowledge must make an enormous difference in expenditure. For instance, although our people are fed so well the cost per head is 7s. 1d. per week, which is below the average."

"Then, I see, that especial pains are taken to serve the meals hot to the patients."

"You refer to the number of wagons. We have had them in use for nine years; they were expensive to start with, costing £25 each, but they have not needed any repairs. The assistant matron superintends the carving and serving. Some idea of the quantity of food consumed may be gathered from the fact that 80 (4 lb.) loaves of bread are sent in daily. As you see, we have separate store-rooms for bread, milk, and for meat, for groceries, and, in fact, for almost everything."

### The Home and the Staff.

Having inspected these store-rooms, we proceeded to the Nurses' Home, which is connected with the Hospital by a handsome conservatory, the gift of Sir John Holder. In the Home itself there are 110 bedrooms, each nurse having a separate apartment. Here the sisters, as well as the probationers and staff nurses, have a room of their own instead of one attached to their respective wards. In the nurses' room there is a library of 500 volumes, and each sitting-room contains a piano. The writing and silence-room is comfortably furnished, and every detail, not excluding the provision made to insure the rest of the night nurses being undisturbed, has been well thought out. The

THE NURSES OF THE GENERAL HOSPITAL, BIRMINGHAM—*continued.*

Home Sister is in charge, and presides at the nurses' meals in the dining-room of the hospital.

As we passed through some of the wards I inquired how they are staffed.

"During the night," rejoined the Matron, "there is a staff nurse and a probationer on duty in every ward. Of course they require time for their meals, which they have separately in the ward kitchens, but the ward itself is never left unattended, and the night superintendent is in charge of the whole of the wards. The number of beds occupied is 320; the men's blocks are on the ground and first floor, the women's on the second, and the children's on the first. The large wards contain 24 beds each, there being, in addition, two small wards with one bed each on each floor. The large wards are staffed during the day by a sister and three nurses, except the children's ward, which has an extra nurse."

"How many sisters and staff nurses are there?"

"Sixteen sisters and ten staff nurses. All are fully trained, and some of the former have been here a number of years. We have just pensioned off a sister who had been with us for 36 years. The sisters are on duty until 9 P.M., but they get three hours off one day, three and a half the other day, and once a month they are free from 2 P.M. on Saturday to 9 A.M. on Monday. On Sundays they are alternately off duty from 10 A.M. to 1. P.M., and 4.30 to 10.30 P.M."

"What about the staff nurses and probationers?"

"They all go into the wards at 7 A.M. and leave at 8.45 P.M. Their off duty varies a little, but averages two-and-a-half hours a day, and on alternate Sundays they have two and six hours off. The staff and assistant (the fourth and third year nurses) have one day a month and the probationers half a day. The night nurses enter the wards at 8.45 P.M. and leave them at 8.45 A.M. They have three hours off duty, with alternate Sundays, from 9.30 A.M. to 1 P.M. and 5 to 8 P.M. With regard to holidays the sisters have four weeks and the nurses three."

"How long has the system of four years' training been in operation?"

"Two years. The system is, however, that probationers who train for four years do not pay a premium, whereas those who sign for three are required to pay 20 guineas. The fourth year is, therefore, an advantage to the hospital and to the nurses. The probationers receive a salary in their second year of £16, and £18 in their third, £20 in fourth. In their fourth year if they have had their certificate they are paid £24, rising by £2 to £28. The sisters start with £30 and rise by £2 a year to £36."

"Is there any help given in the shape of pensions?"

"A number of our nurses belong to the Royal National Pension Fund, and I should be very glad if the hospital itself could be affiliated to the Fund. But although the institution itself dates back over 150 years, the new building has only been opened nine, and we are not yet in a position to do more than grant occasional pensions in the case of very long service."

"Of course the nurses are provided with uniform?"

"For indoors, and I raise no objection to outdoor uniform being worn. It is rather amusing to notice that directly a candidate is accepted as a probationer, she buys out-door uniform; whereas the sisters never think of wearing it. Just the different point of view, you see."

### General and Special Training.

"You might give me, please, some idea of the theoretical teaching."

"In the first year a course of lectures on elementary subjects is given by the matron; courses of eight classes each are held by four of the sisters, each sister taking a class of four or five nurses, and four classes are held by the theatre sister on instruments and on preparation of the theatre for operations. During the second year 15 lectures are given on anatomy, physiology, and bacteriology by a casualty assistant physician, and a surgical casualty officer; four lectures on obstetric and gynæcological nursing by the assistant obstetric officer; a lecture on the Ear and Throat and the Nursing of Ear and Throat cases by the aural surgeon and laryngologist; and a lecture on the Eye and the Nursing of Eye cases by the ophthalmic surgeon. In the third year ten lectures on Medical Nursing are given by an assistant physician and ten on Surgical Nursing by an assistant surgeon. We have also in the third year a course of lectures by an expert on Sick-room Cookery. For these cookery lectures the nurses pay just a trifle, but they are allowed free use of the kitchens, and are provided with materials."

"Your operation work must be on a very large scale?"

"We have on an average daily 10 operations, and on some days as many as 20. There are also a good many at night, which, as I pointed out to you, take place in the large theatre, which at night is in charge of the night sister, who always has a nurse to get it ready. The sister in charge of the theatre during the day is always assisted by a permanent staff nurse and two probationers."

"Of your many features, the out-patients' department strikes me as one of the most remarkable."

"It is certainly a very important one. We have about 62,000 cases a year, and a great many casualty patients come during the night. There is a sister in charge with two permanent staff nurses and two probationers, and a night nurse is always on duty."

"How often do you move your nurses into different wards?"

"During the first, second, and third years they are moved every three months. With regard to special experience there is a masseuse, a fully-trained nurse, who always has a probationer for three months, so that every year four of our probationers have the privilege of going up to London to pass their massage examination. Then our nurses have two months or six weeks in the gynæcological ward, but they are only sent into that ward when they know something of surgical work."

"Have you any Eye beds?"

"A few, but most of the experience of the nursing of ophthalmic cases is gained in the out-patients' department. There are a great many accident cases, and of course the number of medical cases is very large. In one respect I think we are unique. The average stay of our medical or surgical cases in the hospital is three weeks. The more chronic cases go to a relief branch, the Jaffray Hospital, three miles out of the city."

### The Relief Branch.

"Is the staff there entirely different?"

"Oh, yes, it is quite distinct. There are 56 beds in the Jaffray Hospital, and they only take chronic cases from us. We are so full here that we are often obliged to have cases on the couches, and the branch is always full."

"I notice that you have the plenum system. Do you find it has advantages?"

"It makes the place very clean, and saves a lot of work in dusting and in fires. Practically, the only fire we have is in the nurses' sitting-room. If we had fires in the wards and all the rooms we should want a great many more servants than we have at present."

"Speaking of cleanness, your corridors supply an objectlesson, and, with your beautiful chapel, considerably add to the dignity of the institution."

"The lining of the walls of the chapel and the fittings were all private gifts, and the memorial windows to different members of the staff were also provided privately."

The League.

"Your latest addition is the issue of your Nurses League Journal?"

"The League itself was formed at the beginning of this year. All nurses trained here since this hospital was built are eligible for membership, and there are about 150 members. I think it is a very good thing to have such an organisation. It certainly keeps me in touch with all the nurses that have been trained under me, and enables me to know what they are doing. At the meeting on the 1st May there was a large attendance, many coming from a distance, while many more wrote to say that they hoped to be able to come to the next meeting. A journal is issued once a year, and I think that the League and its organ will do much to promote unity amongst the past and present nurses of the Birmingham General Hospital."

Previous Training.

"You have, I think, been Matron of the hospital from the time this building was completed, and came from London here?"

"Yes, I came from the Park Hospital, Hither Green, which I opened, and of which I was matron for 12 months. Previously, after my training at the Royal Southern Hospital, I was sister at Cardiff Infirmary, sister, and subsequently night superintendent, at Monsall Fever Hospital, Manchester, matron of a small Hospital at Devizes, where I obtained some valuable housekeeping experience, matron of Middlesbrough Hospital, and matron for four years of Homerton Fever Hospital. I have found my fever experience, under the Metropolitan Asylums Board, extremely valuable in carrying on my subsequent work."

"Do you object to take probationers who have had previous experience?"

"No, I rather like to have them, always on the condition that they have a good record. They must do something to fill up their time before their general training, and I do not see why they should not profit by children's or fever training. On this point, however, I may say that last year the Board reduced the age of admission here from 23 to 21, providing that a candidate is found on examination to be physically fit. That, I think, is my last word, except that I should like to say that I am in favour of registration, not because it would at all benefit the leading training schools, but because, in my opinion, it would help the smaller hospitals. It would also, I believe, have the effect of weeding out the unsuitable women, and would thus make a great difference to the public."

### Isthmian Canal Service in Panama.

BY AN AMERICAN NURSE.

Since the United States Government has opened hospitals in Panama the terms on which nurses are engaged may interest readers of The Hospital. On applying to the Civil Service Commission in Washington I was informed that a schedule of examinations is issued twice yearly in the middle of January and July. The age limit is twenty to thirty-five years. Applicants must be graduates of schools for trained nurses having at least a two years' course, or have served at least one enlistment in the hospital corps of the United States Army, and must file with their applications copies of testimonials showing their qualifications as nurse based upon experience since graduation. Hospital experience in connection with the treatment of tropical diseases will be given special credit.

Subjects for examination are anatomy and physiology, hygiene of the sick-room, general nursing, surgical nursing, obstetrical nursing, and experience in nursing. The salary is $720 per annum with return passage from port of embarkation. Leave of absence is granted at the end of every twelve months of services rendered, at the rate of six weeks; and at any time after eight months' service at the discretion of the head of the department; this leave of absence may be accumulative for two years. In the event of illness, sick leave is granted with full pay for one month upon the certificate of an authorised physician of the health department in the Canal zone.

In all authorised cases of leave of absence the nurse is entitled to return expenses on the steamers of the Panama Railroad and Steamship Company operating between New York and Colon. Free medical and hospital attendance in case of illness is provided. Each applicant for the Isthmian Canal Service is required to submit to the examiner on the day she is examined a photograph of herself taken within three years, which is filed with examination papers for purposes of identification in case of appointment. Baggage tc the amount of 200 lbs. is allowed free of charge.

Having obtained so much information the applicant sends for Form 1312, which appears somewhat appalling; there are twenty-two personal questions to be answered relative to health and experience in both hospital and private nursing, with the usual questions—age, height, and weight.

Next comes the medical man's certificate, which is very inclusive in every detail of sight, hearing, pulse, heart, and pulmonary sounds; then a notary public has to fill in his part of Form 1312, and lastly two vouchers who have known the applicant for at least six months have their questions to answer.

The final item of the form is the names and addresses of five referees who can answer for the good conduct of applicant for the previous five years.

With a sigh of relief this form is at last completed and must be returned at once to Washington. If approved then the applicant is informed that she may attend examination at whatever place she has named in Form 1312. For female nurses seven hours are allowed, and six hours for male nurses, as in their case obstetrics is omitted. The average number of questions is six in each subject.

Here are examples :—

Describe the gastric juice;

Describe the lungs;

What care in selecting room for contagious case?

How disinfect patient and room?

What bones form the knee-joint?

What hospital or hospitals were you trained in, give dates of each appointment, dates of diplomas, and name of superintendent of nurses?

What symptoms would indicate sepsis during puerperal state?

How give child first bath, and how dress cord?

How sponge typhoid patients?

How give bath to patient in bed?

Have you ever nursed in the tropics? when, where, for how long? give dates of each appointment and nature of duties.

What do for sunstroke while waiting arrival of physician?

No Government post can be procured in the United States,

**ISTHMIAN CANAL SERVICE IN PANAMA**—*continued.*

Panama, the Philippines, Porto Rica, or India, unless the applicant takes the Civil Service examination. It will be seen that it is not the length of time spent in a hospital that is taken into consideration, but how the nurse used her time and brains during training.

The system of training was raised from two to three years in the United States in 1900. This, however, is not considered a means of crushing a one and two years' nurse out of the profession. Whether possessing a one, two, or three years' certificate, the nurse who acquits herself well at the examination is the one who gets the credit and the vacant post.

For the Government posts in the United States all applicants must be American citizens. After the examination is held the papers are arranged by subjects, and forwarded under seal to the Commission at Washington, where they are examined and rated by several examiners, then redistributed and the first rating reviewed by other examiners. When all are rated then those of each competitor are brought together for the first time and the average percentage determined. As each subject is rated by one examiner and reviewed by another it will be seen that impartiality, accuracy, and uniformity are secured in the work. The lowest percentage of rating, so as to pass, is 70 per cent. A notice of the rating is sent to each applicant whether they fail or pass. The papers are not examined and rated by local authorities, but are sent direct to Washington.

### Our Summer Holiday.

BY A NURSE.

We were anxious to spend a week of our precious summer holiday together, my friend and I. The question was, Where should we go? We wanted pretty country, sea air, plenty to interest us, but not to pay more than £3 10s. each.

Various friends were consulted, many of whom were sceptical as to our success in finding such an ideal place.

However, we decided to go to Minehead, in North Somerset, and were fortunate in obtaining nice clean rooms—sitting and double bedroom—with board, for 25s. a week each, in the principal street of the town. We found Minehead quite a tiny place on a bay, sheltered from the north-west by a high hill, from the top of which a splendid view is obtained of the country as far as the Quantock Hills.

We travelled from town by an excursion train, leaving Paddington at 7.50 A.M. on a Thursday in the middle of July, returning from Minehead on Friday week. The journey was by no means uncomfortable, taking about five hours, and as it was early in the summer the train was not crowded. We found the country round Minehead delightful—so many pretty walks over the hills bordering on Exmoor. I should not recommend anyone to take bicycles, for though the main roads are good, they are very hilly, and the prettiest country is reached by footpaths. Char-a-bancs and coaches run every day to different places of interest, but, being good sailors, we preferred the steamer trips as being less expensive. After breakfast we bathed each day; fortunately the tide was high in the morning during our stay, so that we were able to have our dip before starting on our excursions. Owing to the shallow shore one can only bathe two hours before and after high water.

The steamers call about eleven at Minehead pier. We usually took meat sandwiches with us, had tea at the various places we visited, and returned to a meat supper. Our first trip was to Clovelly—such a lovely day, sunny and breezy. En route we had an excellent view of the beautiful North Devon coast, passing Lynmouth, Ilfracombe, and across Barnstaple Bay to the quaint little village of Clovelly, with its main street winding up between the picturesque cottages, dotted up the cleft in the cliffs, which are covered to the water's edge with lovely woods. We spent three hours there, walking on the beautiful "Hobby Drive," and exploring the hills above, from which we had a magnificent view of the sea, and then did the return trip in the cool evening air, with the sun sinking right down "into the sea."

Next day our friendly steamer dropped us at Lynmouth, where we spent the whole day, rambling up the wooded Lyn valley, where the river rushed and foamed over mossy rocks and stones, with numberless tiny waterfalls, till we reached the "Watersmeet," where we rested in the shade near the big waterfall, then back to Lynmouth for tea, and up the little cliff railway to Lynton, and by a path round the rugged cliff to Castle Rock and the Valley of Rocks, which looked weird and wild in the evening mist.

Our next excursion was to Tenby, across the Channel. Four hours' steaming (calling at the Mumbles and giving us a good view of Worms Head and other points of interest on the Welsh coast) brought us to this pretty town, with its north and south sands, interesting old church and castle ruins. Our stay here was necessarily short, as the boat returned at five, but we enjoyed another splendid sunset. Then we determined to go for a coach drive over the hills to Porlock, a little fishing village eight miles away, which we found very fascinating with its many winding corners and picturesque cottages covered with creepers and gay little gardens around. From Porlock we proceeded by a footpath through beautiful woods skirting the seashore for nearly two miles in search of Culborne church, which we at last discovered shut in by hills and trees, standing by itself in a deep glen. This church, which might seat forty people, is called the smallest in the West of England; the tiny black oaken pews hold three persons, and there is an old-fashioned square pew supposed to be 500 years old.

Our last sea-trip was to Ilfracombe.

The remaining day was spent at Dunster, two miles from Minehead, where we were shown over the grounds and part of the castle, which was besieged in the time of Cromwell. There is a curious old yew hedge 60 feet high in the grounds, and the views from the castle walls are very fine across the Channel to Cardiff, while inland one sees Dunkery Beacon, the highest point of Exmoor. A walk through the village brought us to a curious old building in the Market Square, originally used as a yarn market; many quaintly built cottages; an old water-mill mentioned in Domesday book; while the church is very interesting, with its many side chapels and beautifully carved oak screen.

There were other excursions by steamer to Clevedon, Weston-super-Mare, Chepstow, etc., and coach drives to the Doone Valley, Dulverton, and many other places, which we should have enjoyed had time and money permitted. Feeling quite refreshed by the bracing sea air, we returned to town very sunburnt, having thoroughly enjoyed our cheap and delightful holiday in Lorna Doone's country. Our expenses were as follows:—

| | £ | s. | d. |
|---|---|---|---|
| Board and lodging for one week and one day | 1 | 8 | 6 |
| Railway fare return from London ... ... | | 15 | 0 |
| Luggage in advance ... ... ... ... ... | | 2 | 0 |
| Fare to Clovelly ... ... ... ... ... | | 3 | 6 |
| Fare to Lynmouth ... ... ... ... ... | | 2 | 0 |
| Fare to Tenby ... ... ... ... ... ... | | 3 | 6 |
| Fare to Porlock ... ... ... ... ... ... | | 3 | 0 |
| Fare to Ilfracombe ... ... ... ... ... | | 2 | 6 |
| Tea at each place for six days ... ... ... | | 4 | 6 |
| Sundry expenses, such as pier tolls, tips, guides' fees, etc. ... ... ... ... ... | | 5 | 6 |
| Total ... ... ... ... ... ... | £3 | 10 | 0 |

### Bedside Words.

BY A NURSE.

There is one aspect of nursing that should have its rightful place in a nurse's equipment—her attitude towards her patients ethically. As no two persons come into close contact without some transmission of feeling, it follows that the sympathy and tact of a nurse can do much for a patient. For people are peculiarly sensitive to impression in the isolation of illness, which sets them for the season outside the hurly-burly of everyday life.

To a nurse new to hospital and fresh from refined surroundings, it seems impossible to obey the wise injunction "put yourself in her place." She views the dishevelled, coarse, or degraded person she may be called upon to succour with repugnance. And yet, to quote once again, this time a man who fought temptation and came off conqueror; "There," said he, remarking on the spectacle of a man on his way to the gallows, "there, but for the grace of God, goes John Bunyan!" So the nurse may look in all tenderness upon the poor creature whose ignorance or sin may possibly have brought about the illness from which she suffers. Given like surroundings, equal temptations, and no better training, would the nurse have emerged more of a victor? Humility will at once make confession of doubt.

No nurse of thoughtful temperament would wish to have it said that, because of her timidity, a soul had passed prayerless into that dark valley which doubting souls fear, and yet which, strong or weak, each must tread alone.

It is, of course, inevitable that a nurse becomes familiar with death; and at first the purely physical aspect of this mystery will demand all her resolution, all her courage. But, the fear or repugnance once overcome, the danger will be lest the awfulness and solemnity of the freeing of a human soul from its earthly bondage be lost sight of.

This does too sadly happen. There are women who cover up the stilled form without a prayer, or even a thought of the significance of this experience for the departed. One likes to think in case so sad it is solely because death has so far left that nurse's own circle unbroken—for

"We comprehend not till the ruthless shadow
Darkens our doorway, claims from us our toll.
Ah, then we feel! and cry God spare us!
Nor take from us the treasured of our soul,
Yea, when Death touches with his icy hand,
Then our hearts fail us, then we understand."

Some will urge that a nurse has no time for such ministration as is here suggested—that she would make mistakes, that experience would be wanting, that she would not know what to say; but the need for thought would make her alive to her own beliefs, which would strengthen and grow less nebulous from such effort. Only a word is needed; and surely the rules of no hospital would forbid the nurse, as she dusted the "locker" day by day, saying the morning text.

Comment is unnecessary, even if time allowed, the words are brief, simple, and direct; and if the patient makes any responsive remark the chaplain is only too glad to learn of those who might welcome his aid.

Some kind hearted, gift-loving friend gave to a hospital many years ago square bags of an ugly check which hung at each bed head. These contained three books, but oh, how seldom used by the patients, who had no habits of prayer or daily reading! The Moody and Sankey they appreciated, but if one saw an open prayer-book or New Testament one looked for the screens which betokened the critical condition of the patient, and knew that a busy nurse had left it so, and would return as time allowed and read prayer or verse to the passing soul. Some weeks after a night watch of this kind a nurse was asked to see a second patient in the ward which she had left on the preceding day. Not remembering the man in the least, she was the more surprised by his request for "something she had read to No. 18 the night he died." Nurse waxed nervous, for this work was an effort to her; but verse after verse was repeated fruitlessly until the musical words from the prophet were recalled, "Ho, every one that thirsteth come ye to the waters." The man was an invalided soldier; he had come home to die, from a dry and thirsty land, and the words were very pregnant to him. Nurse sent a message to the chaplain with a thankful heart; for though the reading had seemed to fall on deaf ears, yet another wakeful patient had heard and pondered. There is between a nurse and her every patient a "point of contact" if she will only strive to discover it, and that it is well worth the effort is exemplified over and over again, and touched by the love of Christ . . . compassion will gain for us its true meaning. We shall minister to the weak and the erring—and those disabled by illness—not in condescending pity, but as enabled to share evils which are indeed our own."

### Saving Herself Trouble.

I had been out in my district all day, and was just sitting down, tired out, to a cosy tea, when a small boy was admitted demanding to see nurse.

"What is it, Tommy?" I asked.

"If you please, will you come to our 'ouse as quick as you can?" he said.

"What is the matter? Who is ill?" I asked again.

"'Er said will you come as quick as you can?" he repeated, and I felt I must not waste time.

Stifling my regrets for my tea, and wheeling my bicycle, I followed my small guide, who seemed unable to describe the whereabouts of "our 'ouse," except by vague pointings.

"Our baby's swallered a 'apenny," at last he volunteered on the way.

In about twenty minutes we arrived at a cottage, in the doorway of which a big girl was nursing the baby. The latter, on the whole, appeared very well, though very tearful from constant slapping on the back, which, however, had failed to recover the lost coin.

"Where is your mother?" I asked the girl.

"Upstairs, along o' granny," she replied vacantly; "yer can go up."

"Never mind," I said, "I won't disturb your mother now, I will tell you what to do, it is quite simple, and you can tell your mother from me." I gave her the necessary directions as to the baby's treatment, and, promising to call again next day, mounted my bicycle and rode quickly home.

Here I was again hindered for some minutes, and had just comfortably settled down to my meal—this time I had really accomplished the making of the tea, when the same small boy appeared, with the same remark, "If you please, will you come to our 'ouse as quick as you can?"

"Is the baby worse?" I asked, picturing to myself all the possible and impossible complications consequent on the swallowing of a foreign body.

"'Taint the baby 'er wants yer for," he explained, "'er wants yer for granny upstairs, 'ers ill!"

I am wiser now, and no longer rely upon small boys and bigger girls to give me details about "the patient."

### Everybody's Opinion.

**[Correspondence on all subjects is invited, but we cannot in any way be responsible for the opinions expressed by our correspondents. No communication can be entertained if the name and address of the correspondent are not given as a guarantee of good faith, but not necessarily for publication. All correspondents should write on one side of the paper only.]**

### SPINAL CARRIAGE.

"District Nurse" writes from Elm Villa, Warsash, Hants: Owing to your kindness in inserting my appeal for a spinal carriage for an invalid girl in this district, I am thankful to say I have got a very good one sent me from the Matron, Frascati, Surbiton, Surrey. The carriage will be very much appreciated by the poor invalid.

### THE PRESCRIBING NURSE WE KNOW, BUT WHAT IS THIS?

"Surgeon" writes from the British Hospital, Nazareth: Regarding my remark on the skin grafting by a nurse in East London, and in answer to her reply in your issue of July 7, may I venture to point out that she has, apparently, missed the point of my first letter, although I consider that it was distinctly made. I do not suggest that skin grafting was not the proper treatment: the satisfactory result proves that it was required. But is not skin grafting distinctly the work of the doctor? Can any permission, by whomsoever given, make this operation the legitimate work of the nurse? It is to this question that I look for the opinion of The Hospital, in the columns of which the record of such a professional act has appeared.

[Our correspondent is correct in considering that any nurse who undertakes skin grafting of her own initiative commits a breach of professional etiquette. We cannot credit that any nurse who has been adequately trained would ever think of undertaking a surgical operation of such a nature. If such conduct were to be brought to the notice of the authorities of the nurse's training school where the perpertrator was trained, we have little doubt that the authorities would seriously consider the propriety of suspending her certificate.—Ed. The Hospital.]

### ARMY NURSING.

"J. M." writes: If the Royal Army Medical Corps had no other fault with the Sister than the natural one of man's dislike to woman when placed outside her sex as a master, his case would surely be good enough for change. Reading over your Note the other day, I see you name jealousy as the cause of it all; but surely you are mistaken, for what is there to be jealous about in those over-rated women? No; the fact is, this system of mixed nursing is chiefly the cause. The wonder is that it has gone on so long, without even more fury than now threatens the very extinction of one or the other. For it cannot be supposed for a moment that men will always be content to act as catspaws or semi-apprentices to these far too proud and most exacting ladies. Besides, why are they not named "nurses"? Surely that noble name is good enough; but I rather think it is not dignified enough, and if used would mean, not play, but work. Civil hospitals are examples for the proper working of the nursing work of our Army hospitals, for there no men are required, and women do their duty better and under more natural conditions than can ever be the case under the system in the Army. I am hoping you may therefore see that the trouble is due to other causes than the one you suggest. Moreover, it seems also as if you had no idea that the feud has been since 1884, and that while it never could be discovered that a Royal Army Medical Corps man had killed a Sister, it was, and is, common knowledge (and the records of that time will bear this out) that frequent court-martials have taken place as the result of the Sisters' reports, and hence severe forms of punishment and imprisonment, not to mention desertions, from Netley. Instead of the Sisters being the victims of men's jealousy, they have far too long been allowed to rule, with a thirst for suffering that would make a savage ashamed."

### THE LATE MATRON OF KING'S COLLEGE.

We have received the following letter from Miss Monk which we are very pleased to publish:—

"Miss Monk finding it almost impossible to write separately to each person who so generously contributed to the presentation made to her on July 30 last, desires to express through the papers her deep and lasting gratitude to Lord Methuen, Chairman of the Committee of Management of King's College Hospital, to the members of the committee, to the medical and nursing staff of the hospital, past and present, and to all her other good friends. She sincerely hopes that all who have given her this mark of their personal friendship and esteem will know how precious to her the remembrance of their goodness will always be. It is to her a subject of deepest regret and sorrow that her failing health should have prevented her finishing the work that she had hoped to accomplish for the great cause of our hospitals and the care of our sick. She especially regrets that she should have been unable to continue her services to the committee at this critical period in the history of King's College Hospital, and to see the completion of the removal of the hospital to Camberwell before her retirement. It is also a great sorrow to her to be compelled to sever her connection with Queen Alexandra's Imperial Military Nursing Service, more especially as Her Majesty had recently paid her the further compliment of placing her on the Council of the British Red Cross Society, in both of which works she took the warmest interest."

### Appointments.

**[No charge is made for announcements under this head, and we are always glad to receive and publish appointments. The information, to insure accuracy, should be sent from the nurses themselves, and we cannot undertake to correct official announcements which may happen to be inaccurate. It is essential that in all cases the school of training should be given.]**

Ashton-under-Lyne Workhouse Hospital.—Miss Edith Emma Douglas has been appointed superintendent nurse. She was trained at Crumpsall Infirmary, Manchester, where she has since been sister. She has also been sister, home sister, and assistant matron at Stockport Poor-law Infirmary.

Jessop Hospital for Women, Sheffield.—Miss Janet Campbell has been appointed theatre sister. She was trained at the Victoria Infirmary, Glasgow, and the Samaritan Hospital for Women, at Glasgow.

Leek Poor-law Infirmary.—Miss E. L. Wise has been appointed superintendent nurse. She was trained at Wandsworth and Clapham Infirmary, and has since been charge nurse at Stoke-on-Trent Poor-law Infirmary.

Nurses' Co-operation, Leicester.—Miss Grace Larkum has been appointed matron. She was trained at Lincoln County Hospital, and has since been night sister in the same institution.

Queen Charlotte's Hospital.—Miss Inge Bröchner has been appointed matron of this hospital. She was trained at St. Bartholomew's Hospital and has since been sister of the lying-in ward, night superintendent, and assistant matron at the Lewisham Infirmary. She holds the C.M.B. certificate.

Queen Victoria Jubilee Institute.—Miss Ellinor Smith has been appointed inspector for Wales under Queen Victoria's Jubilee Institute for Nurses. She received her hospital training at Sunderland Infirmary, and was trained in district work by the Scottish Branch of the Queen's Institute, holding posts as Queen's nurse in Dundee and Annan; she was then appointed charge nurse to the Notts Nursing Federation, and in 1902 became superintendent of the Somerset County Nursing Association.

St. Mary's Hospital, Manchester.—Miss Kate Farmer has been appointed sister. She was trained at Bagthorpe Infirmary, Nottingham, and has since been staff nurse and charge nurse of maternity wards at the Women and Children's Hospital, Leeds.

Salop Infirmary, Shrewsbury.—Miss M. Multon has been appointed sister at this Infirmary. She was trained at the Metropolitan Hospital, Kingsland Road.

Sculcoates Union Infirmary, Hull.—Miss Lena Driver has been appointed charge nurse. Trained at St. Mary's (Islington) Infirmary, where she was afterwards staff nurse, she has since been sister at St. John's Infirmary, Hampstead.

South Eastern Hospital, New Cross, S.E.—Miss S. Williams has been appointed charge nurse. She was trained at the North Riding Infirmary, Middlesbrough, and afterwards acted temporarily as sister in the same institution. She was also for a short time sister at the Grimsby and District Hospital in charge of the male medical and children's wards, also of the out-patients' department.

Union Hospital, Fir Vale, Sheffield.—Miss Agnes Hart has been appointed sister to this hospital. She was trained at the Toxteth Park Infirmary, Liverpool, where she was charge nurse. She held the appointment of night superintendent at the West Ham Union Infirmary, and has since been occupied in private nursing.

Union Hospital, Fir Vale, Sheffield.—Miss Theresa Woodford has been appointed sister at the above institution. She was trained and acted as sister at the Mill Road Infirmary, Liverpool, and finally was private nurse in Stafford.

### The Nurses' Bookshelf.

The Twentieth Century New Testament. A translation into Modern English made from the Original Greek Westcott and Hort's Text. Revised Edition. (London : Horace Marshall and Son. America : The Fleming H. Revell Company, New York and Chicago. 1904. All rights reserved. Price 1s. 6d.)

This is a translation "into the English of our own time" which, though somewhat colloquial and grating in many parts, will serve to elucidate many obscure Biblical contexts to those who are not much accustomed to read the Scriptures closely. Colloquial vernacular language is not always the most suitable for higher themes, and tends to give an air of flippancy when seriousness is required. Some of the amendments are very weak. Thus, "stroke of paralysis" is scarcely as good as the simple "palsy"; "prostrated with fever" is not much simpler than "sick of a fever." There is something jarring when for "birds of the air have nests" one reads "and wild birds have their roosting-places," or for the "herd of swine" in the parable a "drove of pigs" is substituted. "It is not those who are in health that need a doctor, but those who are ill" is not nearly so English as "They that are whole need not a physician, but they that are sick"; and at the healing of Jairus's (Jaeirus in the present volume) daughter, instead of the homely, sympathetic, and dignified language, "Give place : for the maid is not dead, but sleepeth," we have a brusque, hustling dispensary doctor style, "Go away, the little girl is not dead; she is asleep." Similarly, in the Parable of the Sower, "The birds came and ate it up" is no great improvement on "devoured it"; and "brambles" is by no means synonymous with the "thorns" of the old version. The general impression made by this translation is somewhat akin to that produced in an Englishman's mind on reading a French translation of Shakespeare. The sentiments are good, but the diction has lost its dignity and the language shorn of its grandeur, and thereby rendered less sublime. Still, for purely interpretational requirements the translation before us is of immense value, and may be looked upon as a key to many passages not readily understood. For teachers, for Bible readers, and for the clergy the volume has many instructional features. The translation has been a labour of love, is thoroughly sincere and genuine, and deserves the highest praise and appreciation.

Lectures on Midwifery for Midwives. By A. B. Calder, M.B., M.R.C.S. (London : Baillière, Tindall, and Cox, 8 Henrietta Street, Covent Garden, W.C. 1906. Size 9 in. by 5¾ in. Pages 274. 13 Plates, each containing a series of figures (in all 153). Price 5s. net.)

This book, as its title implies, consists of a series of lectures on "Midwifery for Midwives," which many of the author's pupils have asked him to publish "just in the words they were delivered to the class." Although we have no reason to doubt the accuracy of detail in these lectures, we do not care either for the arrangement of the book or for the style in which the lectures appear. We doubt if these lectures will be as helpful to the midwife as would a work systematically arranged in chapters. We have carefully examined the plates, and the result is disappointing. Many of the illustrations must be unintelligible to the reader; they are, too, for the most part too roughly drawn and too small to be of any practical value. They certainly detract from the value of the work. The book is well got up otherwise, and well printed. We doubt, however, if it will appeal to the general body of midwives and maternity nurses.

Summer Holidays. By Percy Lindley (London : 30 Fleet Street, E.C.).

The author has produced a work in accordance with his usual excellent descriptive style. The East Coast is his theme. Here summer holidays have a wider meaning, as "week ends" become an all-the-year-round part of town life, and the Essex borders and the Suffolk and Norfolk sea-coast is readily reached by the Great Eastern express trains with slip carriages at Colchester, Clacton, Frinton, and Walton. The guide describes the scenery from London eastwards, and splendid views are given of Burnham Thorpe—Nelson's birthplace, Cambridge, Ely and Peterborough Cathedrals, and Walsingham Priory. The different historical points are briefly and interestingly reviewed. Opposite page 36 is the picture of a beautiful expanse through the meadows of which slowly flows the silent Stour, so full of history for the learned and of fish for the sportive, but especially marked out as a place of repose for the weary. We have seen it both with the smile and with the tear, and its expression in both guises is kindly winning and sympathetic. It may be taken as symbolic of Mr. Percy Lindley's entire work.

Nelson's Sixpenny Classics. (London, Edinburgh, Dublin, and New York : T. Nelson and Sons, Limited.)

This series of unabridged works by leading authors, including Dickens, Cooper, Thackeray, Kingsley, George Eliot, and Scott, which Messrs. Nelson have published, bound in cloth at 6d. each, should prove most attractive to any book-lover. The type and paper are good, the size is convenient for reading, and the whole edition should command a large sale. In an age of cheap books we have seen nothing of better value or more attractive at the price than these 6d. classics. We believe the publishers would add to the popularity of the series if they were to arrange to bind the works of each author in cloth of the same colour. This step should make the edition very attractive to humble book-lovers who like to have a small library of their own.

### Notes and Queries.

**REGULATIONS.**

**The Editor is always willing to answer in this column, without any fee, all reasonable questions, as soon as possible.**

**But the following rules must be carefully observed.**

1. **Every communication must be accompanied by the name and address of the writer.**
2. **The question must always bear upon nursing, directly or indirectly.**

**If an answer is required by letter a fee of half-a-crown must be enclosed with the note containing the inquiry.**

*Male Nurses.*

(228) Can you give me information regarding the training of male nurses? I have experience in mental cases.—*Attendant.*

There is no training school for civilian male nurses except at the National Hospital for Epilepsy and Paralysis, Queen Square, Bloomsbury.

*Paralysis.*

(229) Can you tell me of a home, within easy reach of Nottingham, where an old gentleman who is paralysed can receive care and nursing? He could pay up to £2 2s. per week.—*Sunny Side.*

You may find what you require in "Medical Homes." The Scientific Press, 28 Southampton Street, Strand, price 7d., or advertise.

*Consumption.*

(230) Can you help me to get my son, suffering from tuberculosis, into a home for some months?—*Constable.*

We do not know whether your son is suffering from tuberculosis of a joint or of the lungs. If the latter, apply to the Mount Vernon Hospital for Consumption, Hampstead, or to the Brompton Hospital for Consumption. Both have country homes.

*The Red Cross Society.*

(231) Can you give me address of the New Red Cross Society?—*A. G.*

5 York Street Buildings, Adelphi, W.C.

*Stewardess.*

(232) Where can I apply for post as stewardess?—*N. E.*

You can apply to any steamship company for a post as stewardess, but the Royal Mail Steamship Co., Southampton, employ nurse stewardesses.

*Army Nursing in India.*

(233) Where can I apply for particulars about army nursing in India and Royal naval nursing?—*A. N. I.*

For particulars respecting admission to Queen Alexandra's Imperial Nursing Service for India write to the Under-Secretary for India, the India Office, St. James's Park, S.W.; and for particulars respecting admission to Queen Alexandra's Royal Naval Nursing Service to the Director-General, Medical Department of the Navy, Admiralty, 18 Victoria Street, S.W.

*County Council Lecturer.*

(234) Can you tell me how to train as County Council Lecturer. I have two years' training in a cancer hospital. Is that enough?—*H. L.*

You had better apply to the National Health Society, 53 Berners Street; as many women become lecturers without any hospital training at all, more will not be required of you.

*School Matron.*

(235) I am anxious to obtain a post as matron in boys' school. I have had eight months' general training and certificates in Cookery and first-aid. Are these qualifications sufficient. As I contracted scarlet fever in a hospital can I compel the authorities to take me back, or can I claim compensation?—*Dum Spiro Spero.*

Your qualifications should be sufficient in a number of cases. Probably you are a good needlewoman, and not young. As your question respecting your hospital is a legal one a solicitor had better be consulted.

**Handbooks for Nurses.**

| | Post Free. |
|---|---|
| "How to Become a Nurse: How and Where to Train." | 2s. 4d. |
| "Nursing: its Theory and Practice." (Lewis.) ... | 3s. 6d. |
| "Nurses' Pronouncing Dictionary of Medical Terms." | 2s. 6d. |
| "Complete Handbook of Midwifery." (Watson.) ... | 6s. 4d. |
| "Preparation for Operation in Private Houses." ... | 0s. 6d. |

**Of all booksellers or of The Scientific Press, Limited, 28 & 29 Southampton Street, Strand, London, W.C.**

### For Reading to the Sick.

PRAYER AND CONSECRATION.

Lord, what a change within us one short hour
Spent in Thy presence will prevail to make,
What heavy burdens from our bosoms take,
What parched grounds refresh, as with a shower!
We kneel, and all around us seems to lower;
We rise, and all, the distant and the near,
Stands forth in sunny outline, brave and clear.
We kneel, how weak! we rise, how full of power!
Why therefore should we do ourselves this wrong,
Or others, that we are not always strong,
That we are ever overborne with care,
That we should ever weak or heartless be,
Anxious or troubled, when with us is prayer,
And joy and strength and courage are with Thee?

*Archbishop Trench.*

"Every day let us renew the consecration to God's service; every day let us, in His strength, pledge ourselves afresh to do His will, even in the veriest trifle, and to turn aside from anything that may displease Him. . . . He does not bid us bear the burdens of to-morrow, next week, or next year. Every day we are to come to Him in simple obedience and faith, asking help to keep us and aid us through that day's work; and to-morrow, and to-morrow, and to-morrow, through years of long to-morrows it will be but the same thing to do; leaving the future always in God's hands, sure that he can care for it better than we. Blessed trust! that can thus confidingly say, 'This hour is mine with its present duty; the next is God's, and when it comes His Presence will come with it.'"

It matters not what we seem to be to ourselves or others, but only how God looks upon us when we pray to Him. This you may take as the test and proof of anything you say, do, or think; and of the real importance of any event that happens to you: What difference does it make when you come to appear before God in prayer? Will it render you more acceptable or not? Let any one notice each day, there can be no better rule or safeguard, what will render him in his hours of prayer most acceptable with God. There can be no better standard or measure of the real value of all things done for Him.—*J. Williams.*

Almighty and Merciful God, Who art the strength of the weak, and Refreshment of the weary, the Comfort of the sad, the Life of the dying, the God of patience, and of all consolation; Thou knowest full well the inner weakness of our nature, how we tremble and quiver before pain, and cannot bear the Cross without Thy Divine help and support. Help me, then, oh eternal and pitying God to possess my soul in patience, to keep that childlike trust which feels a father's heart hidden beneath the Cross. So shall I be strengthened to endure pain and in the very depth of suffering to praise Thee with a joyful heart.—Amen.

*J. Habermann.*

Christ He requires still, wheresoe'er He comes
To feed or lodge, to have the best of rooms;
Give Him the choice, grant Him the nobler part
Of all the house; the best of all's the heart.

*Herrick.*

## Figures and Tables

**Fig. 18. f1:**